# Artificial Intelligence in Obstetric Anomaly Scan: Heart and Brain

**DOI:** 10.3390/life14020166

**Published:** 2024-01-23

**Authors:** Iuliana-Alina Enache, Cătălina Iovoaica-Rămescu, Ștefan Gabriel Ciobanu, Elena Iuliana Anamaria Berbecaru, Andreea Vochin, Ionuț Daniel Băluță, Anca Maria Istrate-Ofițeru, Cristina Maria Comănescu, Rodica Daniela Nagy, Dominic Gabriel Iliescu

**Affiliations:** 1 Doctoral School, University of Medicine and Pharmacy of Craiova, 200349 Craiova, Romania; alinadica34@gmail.com (I.-A.E.); catalina.ramescu@yahoo.com (C.I.-R.); iuliaberbecaru@gmail.com (E.I.A.B.); 2 Department of Obstetrics and Gynecology, University Emergency County Hospital, 200642 Craiova, Romania; dea_andreea05@yahoo.com (A.V.); ionutdanielbaluta@gmail.com (I.D.B.); ancaofiteru92@yahoo.com (A.M.I.-O.); cristinacomanescu85@gmail.com (C.M.C.); rodica.nagy25@gmail.com (R.D.N.); dominic.iliescu@yahoo.com (D.G.I.); 3 Ginecho Clinic, Medgin SRL, 200333 Craiova, Romania; 4 Research Centre for Microscopic Morphology and Immunology, University of Medicine and Pharmacy of Craiova, 200642 Craiova, Romania; 5 Department of Anatomy, University of Medicine and Pharmacy of Craiova, 200349 Craiova, Romania; 6 Department of Obstetrics and Gynecology, University of Medicine and Pharmacy of Craiova, 200349 Craiova, Romania

**Keywords:** deep learning, artificial intelligence, pregnancy, ultrasound, anomaly scan, fetal heart, fetal brain

## Abstract

Background: The ultrasound scan represents the first tool that obstetricians use in fetal evaluation, but sometimes, it can be limited by mobility or fetal position, excessive thickness of the maternal abdominal wall, or the presence of post-surgical scars on the maternal abdominal wall. Artificial intelligence (AI) has already been effectively used to measure biometric parameters, automatically recognize standard planes of fetal ultrasound evaluation, and for disease diagnosis, which helps conventional imaging methods. The usage of information, ultrasound scan images, and a machine learning program create an algorithm capable of assisting healthcare providers by reducing the workload, reducing the duration of the examination, and increasing the correct diagnosis capability. The recent remarkable expansion in the use of electronic medical records and diagnostic imaging coincides with the enormous success of machine learning algorithms in image identification tasks. Objectives: We aim to review the most relevant studies based on deep learning in ultrasound anomaly scan evaluation of the most complex fetal systems (heart and brain), which enclose the most frequent anomalies.

## 1. Introduction

Congenital fetal anomalies, which cause a high infant mortality rate worldwide, are identified as fetal structural abnormalities at standard morphology ultrasound scans, which involve standard planes of visible organs or body parts [1]. A fetal structural anomaly can be identified on the ultrasound in about 3% of pregnancies, which can range from a minor defect to severe multisystem anomalies [2]. Congenital heart disorders (CHDs) are increasingly diagnosed during pregnancy in developed countries. Prenatal diagnosis of CHDs is helpful in cases with severe abnormalities, such as hypoplastic left heart syndrome, transposition of the great arteries, and total anomalous pulmonary venous. Knowing the diagnosis during pregnancy improves treatment outcomes, quickening postpartum intervention and preserving the long-term neurodevelopment of the newborn [3]. The frequency of fetal central nervous system (CNS) abnormalities is second to cardiac malformations. A precise prenatal diagnosis with ultrasound is crucial for the right postpartum therapy for fetal CNS disorders, which significantly cause in utero mortality and postnatal morbidity [4].

Early fetal ultrasound is now a well-recognized technique for detecting fetal abnormalities and monitoring the evolution or development of intrauterine congenital diseases [5]. However, the Eurofetus study [6] that involved 61 obstetrical ultrasound units from 14 European countries showed that only 55% of significant anomalies were identified before 24 weeks of gestation.

The fundamentals of artificial intelligence (AI) as a discipline were established in the 1950s, under the hypothesis formulated by John McCarthy as “Every aspect of learning or any other feature of intelligence can in principle be so precisely described that a machine can be made to simulate it” [7]. Deep learning (DL) is a part of a more prominent family of machine learning techniques built on artificial neural networks (ANNs). The levels of supervision can vary from unsupervised, semi-supervised, and supervised, all being possible [8].

Although medical errors are the third most significant cause of death in the United States [9], AI can reduce this number by improving interpretation accuracy and reducing workload, which can cause critical details to be overlooked. The information processing and distributed communication nodes in biological systems inspired ANNs [10,11]. ANNs and biological structures like the fetal brain differ in many ways. Mainly, ANNs frequently exhibit static and symbolic behavior, whereas fetal organs exhibit dynamic (plastic) and analog behavior.

Rapid advancements in DL algorithms have made them a powerful tool for examining medical images. Numerous types of deep neural networks effectively handle medical picture segmentation [12]. AI in medical science involves classification, localization, detection, segmentation, and registration of medical images. Convolutional neural networks (CNNs) represent one of the main three types of deep learning algorithms, with remarkable progress in image recognition [13]. 

AI-assisted obstetric ultrasound may automatically identify particular fetal structures based on the gestational age of the pregnancy [14]. Also, AI-based automatic measures and evaluations have been implemented in the last decades to decrease intra- and inter-observer measurement variability and to increase diagnosis accuracy [15]. Moreover, AI progress in recent years enabled the development of AI-based techniques to detect fetal anomalies. We need to remember that AI is based on mathematical algorithms, and the accuracy of the information provided depends not only on the algorithm but also on the quality and quantity of the data [16]. 

Our review aims to highlight the performance of AI detection of normal and abnormal aspects of the most prevalent congenital malformations concerning fetal cardiovascular and central nervous systems.

## 2. Method 

**Figure 1:**
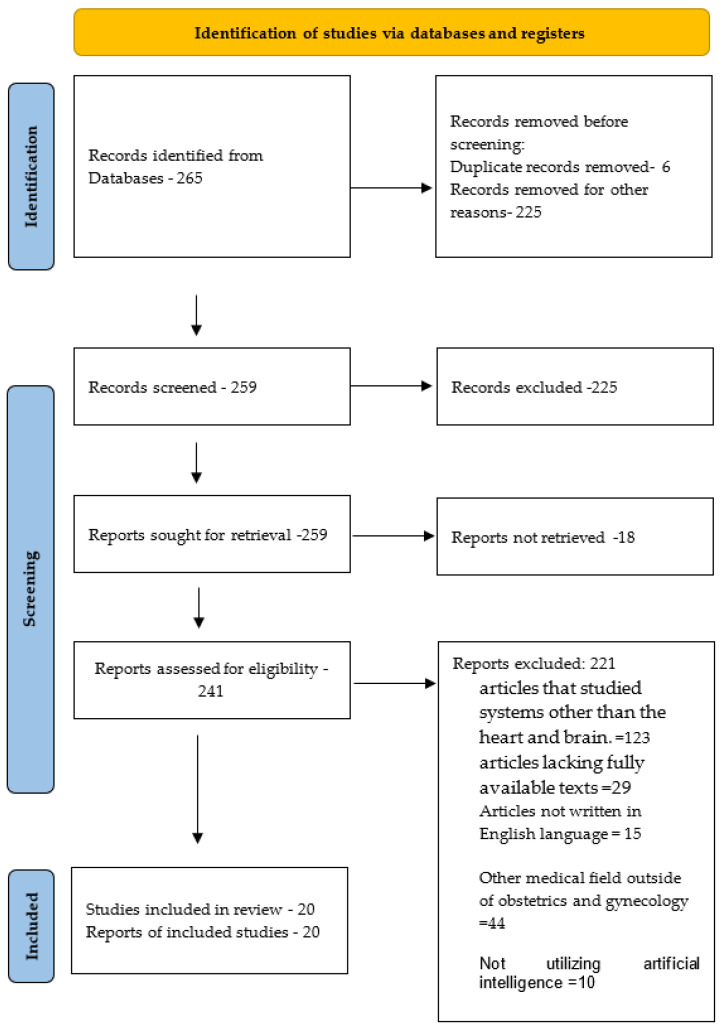
Flow diagram of the method for study selection.

We conducted a search on PubMed, Elsevier, and Scopus using the keywords “deep learning”, “pregnancy”, “Artificial intelligence”, “anomaly scan”, “fetal heart”, “fetal brain”, and “ultrasound”, yielding 265 results from 2015 to 2023. Eligible studies for inclusion had to be in English and focus on discussing the utilization of artificial intelligence in ultrasound and fetal scanning. Two evaluators independently reviewed each study based on the title, abstract, and full text. Studies meeting the selection criteria were included. Each included study underwent assessment and was categorized as 0 = not relevant, 1 = possibly relevant, and 2 = very relevant. Only publications scoring at least 1 point were incorporated into our study. Any discrepancies were deliberated and resolved by a third researcher. Specific exclusion criteria were applied to identify the most pertinent studies. These criteria included excluding studies conducted in languages other than English, those not utilizing artificial intelligence, articles lacking fully available texts, and studies examining systems unrelated to the heart and brain or other medical fields outside of obstetrics and gynecology. After applying the exclusion criteria, we identified 20 relevant articles specifically about the fetal heart and brain along the skull, as shown in Figure 1. 

**Figure 2:**
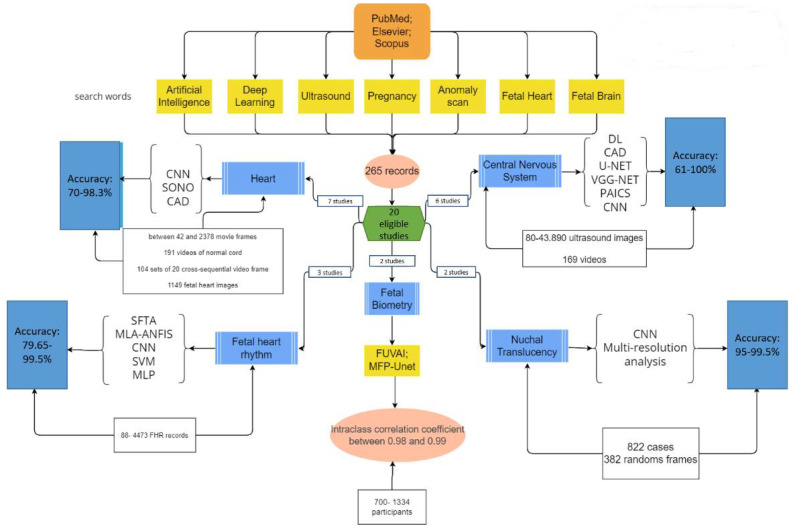
A diagram illustrating the working methods.

Out of the 20 selected articles, 6 addressed the central nervous system, 7 studied the heart, 3 examined the fetal heart rhythm, 2 focused on fetal biometry, and 2 studied nuchal translucency. The working methods are illustrated in Figure 2.

In Figure 2, CNN—convolutional neural network; DL—deep learning; SONO—supervised object detection with normal data Only, AUC—area under the receiver operating characteristic curve; CAD—computer-aided detection; U-NET—network’s U-shaped architecture; VGG-Net—visual geometry group network; PAICS—prenatal ultrasound diagnosis artificial intelligence conduct system; SFTA—segmentation-based fractal texture analysis; MLA-ANFIS—multi-layer architecture of a sub-adaptive neuro-fuzzy inference system; for SVM—support vector machine; MLP—multilayer perceptron; FUVAI—spatio-temporal fetal US video analysis; MFP-Unet—multi-feature pyramid Unet network; MAPSE—mitral valve annular planes systolic excursion; TAPSE—tricuspid valve annular planes systolic excursion; DSC—Dice similarity coefficient; VS—volume similarity; HD95—Hausdorff95 distance; HD—head circumference; BPD—biparietal diameter; AC—abdomen circumference; FL—femur length; HD—Hausdorff coefficient; APD—average perpendicular distance.

## 3. Results

### 3.1. Heart

**Figure 3:**
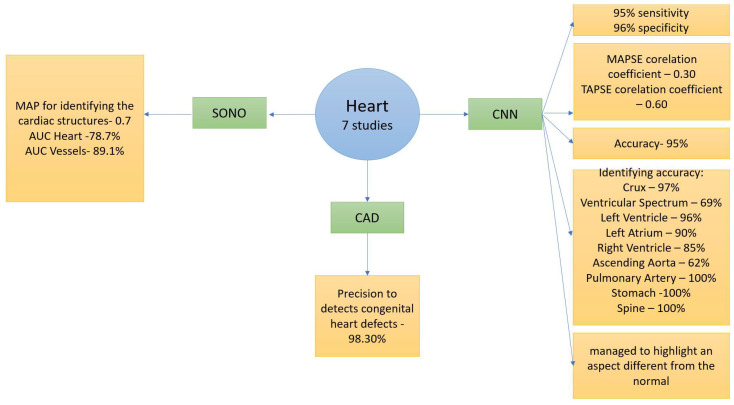
AI studies regarding fetal heart structures.

The fetal heart is a complex organ to analyze and follow because of its nature, continuous movement, and small size. As stated before, congenital heart diseases are the most common [17] fetal malformations. During the first or second trimester scan, sonographers perform an ultrasound anomaly scan as a tool for prenatal diagnosis regarding fetal malformations. Still, the reported detection rates for congenital heart disease remain low [18] Due to these challenges, a novel concept that seeks to integrate AI into ultrasound (US) fetal evaluations to improve the detection rates and overall fetal heart evaluation accuracy has emerged, as shown in Figure 3.

Pregnant women are advised to undergo fetal screening in the second trimester of pregnancy. The fetal heart scan involves examining five standard recommended planes during the cardiac sweep, which enables physicians to diagnose up to 90% of complex congenital heart defects [19].

In a study conducted by Arnaout et al., echocardiographic and second-trimester screening images of fetuses with gestational age between 18 and 24 weeks were analyzed with the help of a variety of neuronal networks, and the authors found that it was possible to distinguish between normal heart development and the presence of inborn cardiac anomalies. The obtained results indicate predictive performances similar to those made by clinical experts, namely, a sensitivity of 95% (95% confidence interval, 84–99%), specificity of 96% (95% confidence interval, 95–97%), and a predictive negative value of 100% [20].

To identify the five screening cardiac plans from fetal ultrasound scans, including three-vessel trachea (3VT), three-vessel view (3VV), left-ventricular outflow tract (LVOT), axial four-chamber (A4C), and abdomen (ABDO) [21], Arnaout et al. [20] used CNNs to categorize the images. Their results showed that the model’s sensitivity is comparable to the physician’s and succeeds at external datasets and lower-quality images. All the images that did not fit the criteria were categorized as non-target images (for example, head, foot, placenta). 

Philip M et al. [22] demonstrated the efficacy of CNNs in the detection and measurement of mitral and tricuspid valve annular planes systolic excursion (MAPSE/TAPSE) for the evaluation of cardiac function with the usage of two separate networks based on the same method, one for mitral valve segmentation and the other for tricuspid valve segmentation. Bland–Altman diagrams were used to analyze differences between measurements made by two experts and the automated method. The TAPSE automatic measurement obtained a correlation coefficient of r = 0.61, while the expert coefficient was r = 0.89. The root mean squared error (RMSE) between the automated and reference measurement systems was 0.14. The R-value for the automated MAPSE measurement was 0.30, for the expert measurement was 0.77, and for the RMSE was 0.18. It was observed that the correlation coefficient, both for the expert and the proposed method for MAPSE, was lower than that of TAPSE. This was due to the rotation movement of MA, which is caused by the circular orientation of the muscle fibers in the left ventricle, which makes MAPSE measurement more challenging than TAPSE measurement [22].

Matsuoka et al. [23] used 2378 movie frames from 51 fetal cardiac screening scans with normal anatomy at 18–20 weeks as the training dataset and 701 movie frames from 28 routine fetal cardiac screening scans as test data. The authors aimed to develop AI to identify the normal position of the heart and aspect of the cardiovascular structures as follows: crux, ventricular septum, right atrium, tricuspid valve, right ventricle, left atrium, mitral valve, left ventricle, pulmonary artery, ascending aorta, superior vena cava, descending aorta, stomach spine, umbilical vein, inferior vena cava, pulmonary vein, ductus arteriosus. The accuracy with which AI managed to identify the heart structures was 97.1% for the crux, 69.3% for the ventricular septum, 96.6% for the left ventricle, 90.6% for the left atrium, 84.8% for the right ventricle, 96.9% for right atrium, 61.9% for the ascending aorta; and 100% for the pulmonary artery, stomach, and spine [23].

Komatsu R et al. [24] used 42 movie frames of a normal heart as a training database from second-trimester scans and identified 18 different plans of the heart and peripheral organs, such as the atrium, ventricle, blood vessels, and stomach. Movie frames with pathologies were introduced in the study, such as Tetralogy of Fallot (TOF) and transposition of great arteries (TGA). The pulmonary artery was not clearly demonstrated in the case of TOF, and the outflow tract and blood vessel detection patterns in TGA were inconsistent compared with a normal fetus. The program failed to highlight the pathology but successfully highlighted the aspects different from normal anatomy, according to the receiver operating characteristic (ROC) curves [24].

In a similar study, Komatsu M et al. [25] proposed a novel architecture of supervised object detection with normal data only (SONO) to detect fetal heart structures and cardiac abnormalities. The correct position of 18 fetal structures was annotated. For this program, 191 videos were used for training, 22 for validation, and 34 for testing. SONO achieved a mean value average precision (mAP) of 0.70 in the testing phase. According to each structure’s average precision (AP), the crux, ventricular septum, ventricles, atria, outflow tract, pulmonary artery, and ascending aorta were well detected. The tricuspid valve, mitral valve, inferior vena cava, pulmonary vein, and ductus arteriosus identification performed poorly in correct detection. To evaluate the detection of abnormal cardiac structures, 104 sets of 20 sequential cross-sectional video frames around a 4CV and a 3VTV obtained from 40 normal and 14 CHD cases were used. In normal cases, the diagnostic components were well-detected and localized, whereas in CHD cases, the detection of fetal structures was very poor. The ROC analyses were used to assess the performance of detecting cardiac structural anomalies in the heart and vessels. The area under the ROC curves (AUC) produced with SONO was 0.787 in the heart and 0.891 in vessels. Therefore, SONO demonstrated the abnormalities more accurately in vessels than in heart chambers.

Nurmaini et al. [26] investigated the use of deep learning-based computer-aided fetal echocardiography for heart standard view segmentation in detecting congenital heart defects. Their study aimed to develop an automated system that can assist medical professionals in detecting congenital heart defects early on. For this purpose, they used 1149 fetal heart images and included three cases of congenital heart defects. The program managed to detect congenital heart defect cases with a precision of 98.30%.

Ungureanu A et al. [18] published a study protocol to develop an automated intelligent decision support system for early fetal echocardiography using deep learning architectures. The authors used ultrasound images from the first-trimester morphology scan using two-dimensional heart loop videos showing a four-chamber view, left and right ventricular outflow tracts, and a three-vessel view. The sample videos were divided into training (60%), validation (20%), and test sets (20%). The primary outcome of their study was an Intelligent Decision Support System (IS) that can assist early-stage sonographers in training for the accurate detection of the four first-trimester cardiac key planes. Another important outcome was an increase in satisfactory heart key-plane evaluations by inexperienced and newly trained sonographers in first-trimester scans. It also resulted in a reduced rate of diagnosis discrepancies between evaluators with different experiences. The study offers the first standardized AI method for fetal echocardiography weeps in the first trimester of fetal heart anomaly detection.

In contrast to previous studies that used AI in the second trimester of pregnancy, Stoean et al. [27] used CNNs in the first trimester of pregnancy and were able to identify four key planes for fetal heart assessment in the first trimester of pregnancy (the aorta, the arches, the atrioventricular flows, and the crossing of the great vessels) with 95% accuracy.

### 3.2. Brain and Skull

**Figure 4:**
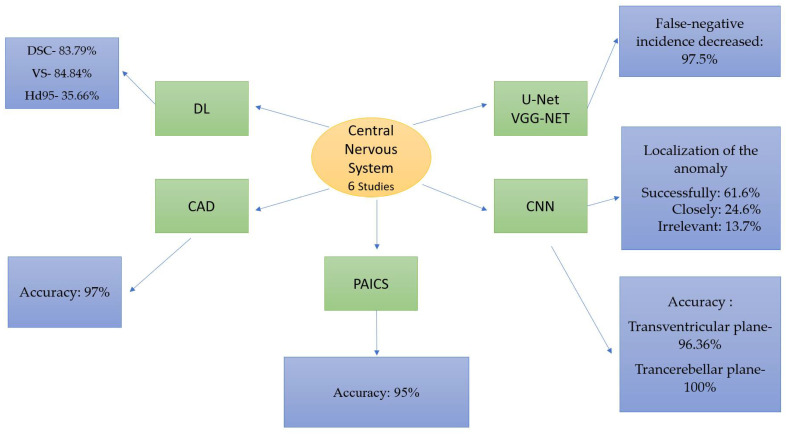
AI studies regarding central nervous system anatomy.

Central nervous system abnormalities are some of the most common congenital fetal malformations, with an incidence rate of 1% [28]. Examining the fetal cranium in standard reference plans, i.e., transventricular, transcerebellar, and transtalamic, represents an essential part of the second-trimester anomaly scan [29,30] Figure 4.

The progress of AI-assisted ultrasound diagnosis enabled a 92.93% accuracy in detecting fetal morphology standard planes; therefore, AI was expected to become an alternative screening method for central nervous system fetal malformations [31].

Huang et al. [32] investigated the use of deep learning algorithms for segmenting brain structures imagined with fetal MRI. Their study provides an accurate and efficient method for brain tissue segmentation in fetal MRIs, which is essential for quantifying the presence of congenital disorders. Manual segmentation of fetal brain tissue is cumbersome and time-consuming, so automatic segmentation can significantly simplify the process. The group analyzed 80 fetal brain MRI scans at gestational ages from 20 to 35 weeks. A 6:1:1 ratio was used to divide the dataset into training, validation, and test sets. Dice accuracy, sensitivity, and specificity were used to evaluate the method objectively. The results indicated an average Dice similarity coefficient (DSC) of 83.79%, average volume similarity (VS) of 84.84%, and average Hausdorff95 distance (HD95) of 35.66 mm. The authors compared their approach with several others and demonstrated the superiority of their method.

Heuvel et al. [33] presented a computer-aided detection (CAD) system for automated measurement of the fetal head circumference (HC) in 2D ultrasound images for all trimesters of pregnancy. The CAD system was tested on an independent test set of 335 photos from all trimesters after being trained on 999 images. A skilled sonographer and a medical researcher personally annotated the test set. The outcomes of 0.98 accuracy on the validation set and 0.97 on the test set demonstrate that the CAD system performs as well as a skilled sonographer. 

Xie B. et al. [34] utilized the first algorithm for prenatal ultrasonographic diagnosis of central nervous system malformations. Xie et al. utilized U-Net for the cranium region segmentation and the VGG-NET network to differentiate the images of the normal and abnormal structures. Thus, the group decreased false-negative results in fetal brain anomalies by 97.5%.

Xie H.N. et al. [35] used DL-based CNNs to classify ultrasound images as normal or abnormal in standard axial neurosonographic planes. Their study included 15.373 typical images and 14.047 abnormal images of the fetal brain, identified correctly using the program in a proportion of 96.9% and 95.9%, respectively. The exact location of the anomaly was identified correctly in 61.6% of the abnormal ultrasound images, closely in 24.6% of the cases, and irrelevantly in 13.7%. Even though these algorithms can perform simple diagnosis, Yaqub et al. [36] assembled a system that identifies septum cavum pellucidum on the transventricular cerebral plane. Baumgartner et al. [37] assembled a CNN-based system, which helped them automatically and in real time determine 13 standard fetal plans, including the transventricular and transcerebellar sections with an accuracy of 96.36% and 100%, respectively. 

Lin et al. [38] developed an AI system based on CNN (PAICS—prenatal ultrasound diagnosis artificial intelligence conduct system) capable of identifying nine different cerebral malformations based on standard, real-time ultrasound examination images, with an average accuracy of 95%. Using the PAICS system reduced the examination time, and the system’s performances were compared with examinations performed by highly experienced practicians.

### 3.3. Fetal Cardiotocography

**Figure 5:**
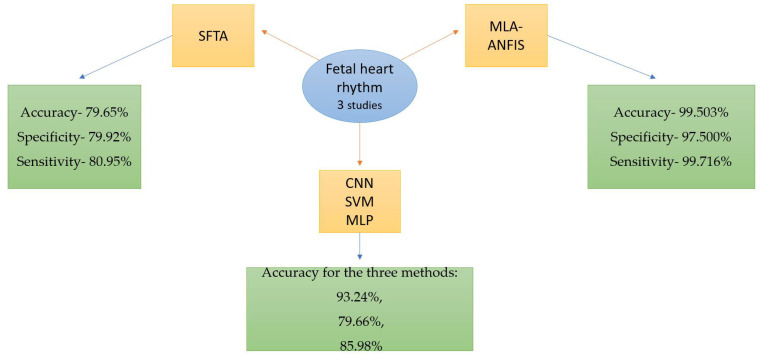
AI studies regarding fetal heart rhythm interpretation.

Cardiotocography (CTG) is crucial for determining fetal status by monitoring the fetal heart rate (FHR) and uterine contractions. The fetal heart rate (FHR) shows remarkable patterns for evaluating fetal physiology and common stress situations, and according to a vast meta-analysis, continuous CTG monitoring is correlated to a 50% decrease in newborn seizures [39] (Figure 5).

Z. Cömert and A. F. Kocamaz used segmentation-based fractal texture analysis (SFTA) to identify normal and hypoxic records. In total, 44 normal and 44 hypoxic fetuses instances were analyzed, resulting in a 79.65% accuracy, 79.92% specificity, and 80.95% sensitivity to distinguish normal and hypoxic fetuses [40].

On a CTG dataset, different topologies of the multi-layer architecture of a sub-adaptive neuro-fuzzy inference system (MLA-ANFIS) were constructed using multiple input features, neural networks (NNs), deep stacked sparse auto-encoders (DSSAEs), and deep-ANFIS models. In a study conducted by Iraji MS, the results obtained with DSSAE were more accurate than other suggested techniques to predict fetal well-being. The method showed a sensitivity of 99.716%, a specificity of 97.500%, and an accuracy of 99.503% [41]. AI has been used with contemporary computer systems to interpret CTG to overcome human limitations, and numerous trials are being conducted in this area. 

CNNs are often used in medicine to create screening systems that automatically aid physicians because of the apparent advantages. Li et al. [42] collected 4473 FHR records and categorized them into three classes: normal, suspicious, and abnormal, based on the electronic fetal monitoring (EFM) system. To improve classification accuracy, the researchers divided the high-resolution 1-dimensional FHR records into ten d-window segments and used CNNs to process the data in parallel. Their study also conducted a comparative experiment. This experiment extracted features from the FHR data using basic statistics. These features were then used as inputs for support vector machine (SVM) and multilayer perceptron (MLP) classifiers. The accuracy of classification was reported for SVM (79.66%), MLP (85.98%), and CNN (93.24%). These percentages represent each classification method’s accuracy rates, with CNN showing the highest accuracy [42]. 

### 3.4. Fetal Biometry

**Figure 6:**
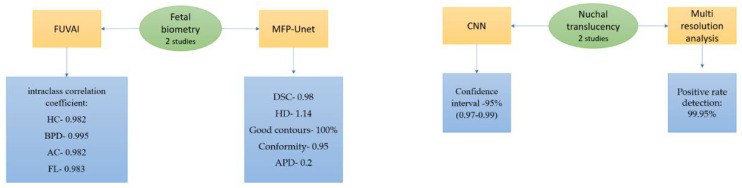
AI studies regarding fetal biometry parameters and nuchal translucency estimations.

Accurate fetal biometric measurements of head circumference (HC), biparietal diameter (BPD), abdomen circumference (AC), and femur length (FL) are used to estimate gestational age (GA) and fetal weight (EFW), which are essential for proper delivery management [43] (Figure 6).

Szymon Płotka et al. [44] used a novel multi-task CNN-based spatiotemporal fetal US feature extraction and standard plane detection algorithm (FUVAI). They used video recordings from 700 pregnancies and compared the FUVAI fetal biometric measurements with those of experienced sonographers. Clinical studies have revealed that errors are less than 15%, which is acceptable in clinical practice [45]. In the same study, the authors found intraclass correlation coefficients (ICCs) between FUVAI and junior readers of 0.982, 0.989, 0.985, and 0.981 for HC, BPD, AC, and FL, respectively, and ICCs between FUVAI and seniors of 0.987, 0.991, 0.987, and 0.986 for HC, BPD, AC, and FL, respectively. Those results show us that FUVAI results are better correlated with senior examinators. For the second and third trimesters of pregnancy, the corresponding values were 0.982, 0.994, 0.980, and 0.981, and 0.982, 0.995, 0.982, and 0.983, for HC, BPD, AC, and FL, respectively, with no notable differences between the second and third trimester of pregnancy [44].

In a study by Oghli MG et al. [46], CNNs were utilized for automatic measurement and segmentation of fetal biometric parameters, including biparietal diameter (BPD), head circumference (HC), abdominal circumference (AC), and femur length (FL) using a multi-feature pyramid Unet (MFP-Unet) network. They trained this algorithm on 1334 subjects and achieved 0.98, 1.14, 100%, 0.95, and 0.2 mm for the Dice similarity coefficient (DSC), Hausdorff (HD), satisfactory contours, conformity, and average perpendicular distance (APD), respectively.

### 3.5. Nuchal Translucency

AI can assist sonographers in automatically identifying the neck region in ultrasound images and measuring the nuchal translucency (NT). Zhang L et al. [47] used CNNs to screen the trisomy 21 by measuring the NT. They enrolled 822 cases in their study, including 550 participants in the training set and 272 participants in the validation set, with a similar mean age. The DL model showed good performance in both sets for trisomy 21 screening with a 95% confidence interval of 0.92–0.95.

Sciortino G et al. [48] proposed a methodology based on wavelet and multi-resolution analysis. They obtained a positive rate of 99.95% concerning nuchal region detection, and about 64% of scans presented an error of 0.1 mm Figure 4.

Table 1 gives an overview and summary of the results obtained from the research we reviewed and contrasts the analysis performed using AI with that performed by conventional sonographers.

### 3.6. Results Summary Table 1

In Table 1, CNN—convolutional neural network; DL—deep learning; SONO—supervised object detection with normal data Only, AUC—area under the receiver operating characteristic curve; CAD—computer-aided detection; U-NET—network’s U-shaped architecture; VGG-Net—visual geometry group network; PAICS—prenatal ultrasound diagnosis artificial intelligence conduct system; SFTA—segmentation-based fractal texture analysis; MLA-ANFIS—the multi-layer architecture of a sub-adaptive neuro-fuzzy inference system; SVM—support vector machine; MLP—multilayer perceptron; FUVAI—spatio-temporal fetal US video analysis; MFP-Unet—multi-feature pyramid Unet network; MAPSE—mitral valve annular planes systolic excursion; TAPSE—tricuspid valve annular planes systolic excursion; DSC—Dice similarity coefficient; VS—volume similarity; HD95—Hausdorff95 distance; HD—head circumference; BPD—biparietal diameter; AC—abdomen circumference; FL—femur length; HD—Hausdorff coefficient; APD—average perpendicular distance.

### 3.7. Results of Syntheses

The predictive values of the AI methods used in the included studies were divided into groups according to the system analyzed and evaluated. The results are summarized in Table 2, Table 3, Table 4, Table 5 and Table 6.

## 4. Discussion 

This review encompasses several articles focusing on using AI in fetal ultrasound assessment. The objective of developing these neural networks is to enhance the process of ultrasound assessment by automating the identification of fetal structures, thereby maximizing the accuracy of the technique and minimizing examination time.

Numerous programs were outlined in the reviewed studies, all of which successfully attained their objectives by achieving accuracy rates exceeding 90% in identifying fetal brain and heart structures or their biometric measurements [27]. These findings have exhibited promising outcomes in enhancing the precision and automation of fetal parameter estimations.

Congenital heart diseases are the most common fetal malformations [4]. The incorporation of AI into ultrasound assessments is directed at enhancing both detection rates and precision. Research studies have showcased the efficacy of AI applications applicable across any gestational age, demonstrating the capability to identify fetal structures as early as the first trimester of pregnancy [14,18]. These studies delineated four established fetal heart assessment key plans and expanded to identify up to nine fetal heart structures in the second trimester [23]. Additionally, a protocol for developing an automated intelligent decision support system for early fetal echocardiography using DL architectures was developed and successfully implemented. The goal is to aid sonographers in identifying correctly the key cardiac planes during the first trimester.

The development of specialized systems, such as those determining various fetal plans, emphasizes the versatility of AI in fetal ultrasound examinations. The potential of AI to enhance prenatal care by providing more accurate and efficient methods for identifying and diagnosing fetal anomalies is evident. These advancements underline the transformative impact of AI on the field, offering a promising avenue for future improvements in fetal healthcare [37]. 

Central nervous system abnormalities are the second most common congenital fetal malformations, with an incidence rate of 1% [28]. AI-assisted ultrasound diagnosis has achieved high accuracy rates of up to 100% in detecting fetal brain standard planes, making it a potential alternative screening method for central nervous system fetal malformations. Notably, specialized software was developed, exhibiting the ability to accurately identify up to 13 fetal brain planes, such as the transventricular plane and the transcerebellar plane, with a remarkable 96.36% and 100% accuracy rate, respectively [37].

Beyond identifying standard fetal planes, AI demonstrated proficiency in distinguishing between typical and abnormal images, effectively pinpointing the location of abnormalities within the fetal brain. AI was able to precisely identify different types of brain abnormalities in real time during ultrasound tests using the PAICS system (95% accuracy) (ventriculomegaly, non-visualization of Cavum septum pellucidum, septum pellucidum, crescent-shaped single ventricle, non-intraventricular cyst, intraventricular cyst, open four ventricles, and mega cisterna magna) [37]. 

Certain programs have utilized cases involving congenital brain anomalies (neural tube defect, holoprosencephaly, lissencephaly, microcephalus, posterior fossa anomaly, spare occupying lesion, intracranial hemorrhage, or ventriculomegaly) as part of the training data, leading to the capability to detect fetal anomalies at an impressive rate of over 96%. Moreover, AI has successfully located an anomaly with an accuracy rate of 61.6% in the cases, closely in 24.6%, and irrelevantly in 13.7% [35].

This approach underlines the efficiency of using AI-based programs as valuable tools for less experienced medical professionals that can significantly support improving diagnostic competence [18].

The use of AI can support sonographers in automatically identifying the neck region in ultrasound images and measuring the nuchal translucency in cases with Down Syndrome. The deep learning model performed well in training and validation sets, achieving a 95% confidence interval by measuring NT [47]. Also, good outcomes were obtained in studies that utilized normal cases for identifying nuchal translucency (99.95% detection of the nuchal region) [48].

Our comprehensive review encompasses diverse AI-based evaluation methodologies, recent studies, their associated advantages and disadvantages, potential obstacles, and the anticipated applications of AI in obstetrics. With this thorough investigation, it becomes evident that AI holds significant promise in prenatal diagnosis [14]. It has the potential to surmount diagnostic challenges, enhance treatment options, and ultimately contribute to improved patient outcomes in fetal medicine.

## 5. Conclusions

AI has seamlessly integrated into various facets of our daily lives and emerged as a pivotal source of innovation in healthcare. It plays a substantial role in supporting clinical decision-making and providing high-quality assistance. AI solutions prove to be highly advantageous, particularly in healthcare domains where professionals such as radiographers and sonographers heavily depend on information derived from images. DL, a subset of AI, excels in image pattern recognition, making it particularly effective for practitioners relying on image-based data for diagnosis and decision-making in healthcare settings.

AI-assisted ultrasound diagnosis addresses certain limitations associated with traditional ultrasound examinations. The substantial progress made in recent years, coupled with enhanced capabilities in detecting prenatal fetal malformations, positions AI as a prospective adjunct or alternative screening method for identifying fetal anomalies. This includes the assessment of complex systems like the brain and heart.

Studies highlight AI’s potential in accurately detecting heart structures. AI, particularly CNNs, effectively distinguishes normal development from cardiac anomalies, with studies showing comparable and predictive performances to experts. 

AI technologies, such as DL algorithms and CNNs, have demonstrated impressive accuracy in identifying brain planes and structures and automated fetal head biometry measurements. Also, comparable performance to the skilled sonographers in anomaly detection and a reduction in false-negative results in diagnosing fetal brain anomalies were obtained.

The development of specialized systems, such as those determining various fetal plans, emphasizes the versatility of AI in fetal ultrasound examinations. The potential of AI to enhance prenatal care by providing more accurate and efficient methods for identifying and diagnosing fetal anomalies is evident. These advancements underline the transformative impact of AI on the field, offering a promising avenue for future improvements in fetal healthcare. 

## Figures and Tables

**Table 1:** Results summary.

Authors	Method	Objective	Pregnancy Trimesters	Results
Arnaout et al. [20]	CNN	Heart	Second	Sensitivity to distinguish normal heart development—95% Specificity to distinguish normal heart development—96%
Philip, M. et al. [22]	CNN	Heart	Second	MAPSE correlation coefficient = 0.30 TAPSE correlation coefficient = 0.61
Matsuoka et al. [23]	CNN	Heart	Second	The accuracy with which AI managed to identify the heart: Crux—97.1%; Ventricular septum—69.3%; Left ventricle—96.6%; Left atrium—90.6%; Right ventricle—84.8%; Ascending aorta—61.9%; Pulmonary artery—100%; Stomach—100%; Spine—100%.
Komatsu R et al. [24]	CNN	Heart	Second	Managed to highlight an aspect different from normal
Komatsu M et al. [25]	SONO	Heart	Second	Median average precision for identifying the cardiac structures—70% AUC heart—78.7% AUC vessels—89.1%
Nurmaini et al. [26]	CAD	Heart	Second	Precision—98.30%
Stoean et al. [27]	CNN	Heart	First	Accuracy—95%
Huang et al. [32]	DL	Brain	Second Third	DSC—83.79% VS—84.84% Hd95—35.66%
Heuvel et al. [33]	CAD	Brain	All trimesters	Accuracy: 97%
Xie B. et al. [34]	U-Net VGG-NET	Brain		False-negative incidence decreased by 97.5%
Xie H.N. et al. [35]	CNN	Brain	Second Third	Localization of the anomaly Correctly—61.6% Closely—24.6% Irrelevant—13.7%
Baumgartner et al. [37]	CNN	Brain	Second	Accuracy of identifying: Transventricular plane—96.36%. Trancerebellar plane—100%.
Lin et al. [38]	PAICS	Brain	Second Third	Accuracy for identifying different cerebral malformations—95%
Z. Cömert and A. F. Kocamaz [40]	SFTA	Fetal heart rate	Third	Accuracy—79.65% Specificity—79.92% Sensitivity—80.95%
Iraji MS [41]	MLA-ANFIS	Fetal heart rate	Third	Accuracy—99,503% Specificity—97.500% Sensitivity—99.716%
Li et al. [42]	CNN SVM MLP	Fetal heart rate	Third	Accuracy of the three methods: 93.24%; 79.66%; 85.98%.
Szymon Płotka et al. [44]	FUVAI	Fetal biometry	Second Third	Intraclass correlation coefficient: HC—0.982. BPD—0.995. AC—0.982. FL—0.983.
Oghli, M. G. et al. [46]	MFP-Unet	Fetal biometry	Second	DSC—0.98 HD—1.14 Good contours—100% Conformity—0.95 APD—0.2
Zhang L et al. [47]	CNN	NT	First	Confidence interval—95% (0.92–0.95)
Sciortino G [48]	Multi resolution analysis	NT	First	Positive rate of detection: 99.95%

**Table 2:** Cord studies—synthesized records.

Arnaout et al. [20]	Boston	107,823 images	Gestational age between 18 and 24 weeks	CNN	Sensitivity of 95% Specificity of 96% Predictive negative value of 100%
Philip M et al. [22]	New South Wales	95 participants	Mean gestational age of 30.7 Gestational age between 22.9 and 38.0	CNN	RMSE for TAPSE—0.14 RMSE for MAPSE—0.18
Matsuoka et al. [23]	Japan	2378 movie frames from 51 fetal cardiac screening scans used as the training dataset 701 movie frames from fetal cardiac screening used as test data	Gestational age between 18 and 20	CNN	The accuracy with which AI managed to identify the heart was between 61.9 and 100%
Komatsu R et al. [24]	Japan	42 movie frames for database	Second trimester	CNN	Managed to highlight an aspect different from normal
Komatsu M et al. [25]	Japan	191 videos of normal cord used for training 22 videos used for validation 34 videos used for testing	Second trimester	SONO	Mean value average precision (mAP) of 0.70
Komatsu M et al. [25]	Japan	104 sets of 20 sequential cross-sectional video-frames	Second trimester	SONO	AUC for heart—0.787 AUC for vessels—0.891
Nurmaini et al. [26]	Indonesia	1149 fetal heart images	Second trimester	CAD	Precision: 98.3%
Stoean et al. [27]	Romania	7251 fetal heart images	First trimester	CNN	Accuracy: 95%

**Table 3:** Brain studies—synthesized records.

Huang et al. [32]	China	80 fetal brain scans	20–35 gestational age	DL	Dice coefficient—83.79% VS—84.84% Hd95—35.66%
Heuvel et al. [33]	Netherlands	999 images for the training set 335 images for test data	All trimesters	CAD	Accuracy on the validation set—0.98 Accuracy on the test set—0.97
Xie B. et al. [34]	China	13.350 images	18–32 gestational weeks	U-net VGG-Net	False-negative incidence decreased by 97.5%
Xie HN et al. [35]	China	13.373 normal pregnancies 14.047 abnormal pregnancies	Second trimesters	CNN	Located lesions: Precisely in 61.6%; Closely in 24.6%; Irrelevantly in 13.7%.
Baumgartner et al. [37]	UK	2694 ultrasound examinations	18–22 gestational weeks	CNN	Accuracy to identify the correct plans between 96.36% and 100%
Lin et al. [38]	China	43.890 ultrasound images 169 ultrasound videos	18–40 gestational weeks	PAICS	Accuracy to identify the correct plans—95%

**Table 4:** Fetal heart rhythm studies—synthesized records.

Z. Cömert and A. F. Kocamaz [40]	Turkey	44 normal fetuses 44 hypoxic fetuses	Third trimester	SFTA	Distinguished normal and hypoxic fetuses with: Accuracy—79.65%; Specificity—79.92%; Sensitivity—80.95%.
Iraji MS [41]	Iran		Third trimester	MLA-ANFIS DSSAEs Deep-ANFIS	Predicted fetal well-being with: Specificity—97.500%; Accuracy—99.503%; Sensitivity—99.716%.
Li et al. [42]	China	4473 FHR records	Third trimester	SVM MLP CNN	Accuracy for classification in three classes: normal, suspicious, and abnormal SVM—79.66%

**Table 5:** Fetal biometry studies—synthesized records.

Szymon Płotka et al. [44]	Poland	700 pregnancies	Second and third trimester	FUVAI	Intraclass correlation coefficient: HC—0.982; BPD—0.995; AC—0.982; FL—0.983.
Oghli, M. G. et al. [46]	Iran	1334 subject	Second	MFP-Unet	DSC—0.98 HD—1.14 Good contours—100% Conformity—0.95 APD—0.2

**Table 6:** Nuchal translucency studies—synthesized records.

Zhang L et al. [47]	China	822 cases	11–14 gestational weeks	CNN	Confidence interval—95% (0.92–0.95)
Sciortino G [48]	Italy	382 cases	FIRST	Multi resolution analysis	Positive rate of detection—99.95%

## Data Availability

Data sharing is not applicable to this article.
